# Cognitive Function of* Artemisia argyi* H. Fermented by* Monascus purpureus *under TMT-Induced Learning and Memory Deficits in ICR Mice

**DOI:** 10.1155/2017/5809370

**Published:** 2017-09-10

**Authors:** Jin Yong Kang, Du Sang Lee, Seon Kyeong Park, Jeong Su Ha, Jong Min Kim, Gi Jeong Ha, Weon Taek Seo, Ho Jin Heo

**Affiliations:** ^1^Division of Applied Life Science (BK21 Plus), Institute of Agriculture and Life Science, Gyeongsang National University, Jinju 52828, Republic of Korea; ^2^Department of Agricultural Processing, Gyeongsangnam-do Agricultural Research and Extension Service, Jinju 52733, Republic of Korea; ^3^Department of Food Science, Gyeongnam National University of Science and Technology, Jinju 52725, Republic of Korea

## Abstract

The cognitive effect of* Artemisia argyi H. *under liquid-state fermentation by* Monascus purpureus* (AAFM), which has cellular antioxidant activity and neuronal cell viability, on trimethyltin- (TMT-) induced learning and memory impairment in Institute of Cancer Research (ICR) mice was confirmed. Tests were conducted to determine the neuroprotective effects against H_2_O_2_-induced oxidative stress, and the results showed that AAFM has protective effects through the repression of mitochondrial injury and cellular membrane damage against H_2_O_2_-induced neurotoxicity. In animal experiments, such as the Y-maze, passive avoidance, and Morris water maze tests, AAFM also showed excellent ameliorating effects on TMT-induced cognitive dysfunction. After behavioral tests, brain tissues were extracted to assess damage to brain tissue. According to the experimental results, AAFM improved the cholinergic system by upregulating acetylcholine (ACh) contents and inhibiting acetylcholinesterase (AChE) activity. AAFM effectively improved the decline of the superoxide dismutase (SOD) level and the increase of the oxidized glutathione (GSH) ratio and lipid peroxidation (malondialdehyde (MDA) production) caused by TMT-induced oxidative stress. The occurrence of mitochondrial dysfunction and apoptosis was also decreased compared with the TMT group. Finally, quinic acid derivatives were identified as the major phenolic compounds in AAFM using ultra-performance liquid chromatography quadrupole-time-of-flight (UPLC-Q-TOF) MS analysis.

## 1. Introduction

*Artemisia argyi* H. is a perennial herbaceous plant belonging to the* Chrysanthemum* family, and it has been used as a drug in traditional medicine for a long time in China, Korea, Japan, and the Russian Far East. Studies on* Artemisia argyi* H. have revealed its various biological functions, such as antidiabetic, antioxidant, anticancer, and anti-inflammatory [1, 2]. These results have proven that* Artemisia argyi* H. is a valuable material, but research using this plant is lacking.

The current study suggests that microbial fermentation may offer an alternative to conventional extraction and hydrolysis methods through enzymatic hydrolysis. This technique can decrease solvent consumption, and it is more environmentally friendly. In addition, it can improve the extraction yield and the quality of the extracts. Sometimes, several microorganisms can enhance the total phenolic content and antioxidant activities of fermented extracts [3, 4]. Phenolic compounds, which are found in over 10,000 different plants, are well known as a source of natural antioxidants and have beneficial effects on human health through their antioxidant capacity [5]. Phenolic compounds of* Artemisia argyi *H. under liquid-state fermentation by* Monascus purpureus* (AAFM) have higher total phenolic contents and antioxidant activity than other strains' fermented products (see supplementary materials available online at https://doi.org/10.1155/2017/5809370).* Monascus purpureus, *one of the strains used for fermentation, has been used in many traditional fermented foods in East Asia, including Korea, China, and Japan.* Monascus purpureus* is a species of fusarium including Ascomycotina, Plectomycetes, Eurotiales, Monascaceae, and* Monascus*. It is taxonomically affiliated with ascomycetes, and it is also called red yeast rice due to the red color of the hypha [6]. In addition, various studies have reported that* Monascus*-fermented products contain bioactive metabolites and that* Monascus*-fermented products are effective for the management of blood cholesterol, diabetes, blood pressure, and obesity and the prevention of cancer development [7]. Therefore, this study was conducted to confirm the ameliorating effect of AAFM on cognitive dysfunction in mice caused by trimethyltin (TMT). TMT is a type of organotin compound that induces neuronal cell death in the central nervous system through strong toxins [8]. The abnormal mechanism of hippocampal neuronal damage caused by TMT is irreversible, and it induces oxidative stress, which causes protein oxidation, lipid peroxidation, DNA damage, and cell membrane injury. Therefore, it is widely used to establish cognitive disorders, such as Alzheimer's disease (AD) mouse models [9, 10].

## 2. Materials and Experimental Methods

### 2.1. Materials

TMT, ascorbic acid, dimethyl sulfoxide (DMSO), 2′,7′-dichlorofluorescein diacetate (DCF-DA), 2′,3-(4,5-dimethylthiazol-2-yl)-2,5-diphenyltetrazolium bromide (MTT), lactate dehydrogenase (LDH) assay kit, superoxide dismutase (SOD) assay kit, and all other chemicals were purchased from Sigma-Aldrich Chemical Co. (St. Louis, MO, USA). Phosphoprotein kinase B (p-Akt; Ser 473; 9271S), BAX (2772S), and anti-rabbit (7074S) and anti-mouse (7076S) antibodies were purchased from Cell Signaling Technology (Danvers, MA, USA). Phospho-c-Jun N-terminal kinases (p-JNK; sc-6254), cytochrome c (sc-13560), p-tau (Ser 404; sc-12952), and *β*-actin (sc-69879) antibodies were obtained from Santa Cruz Biotechnology (Dallas, TX, USA).

### 2.2. Sample Preparation

Hot-air dried* Artemisia argyi* H. is extracted with 10 volume distilled water at 121°C for 30 min. In addition, for manufacture of seed culture, rice (7 g) was put into water (250 mL) for 3 h, and then after removing the water it was sterilized at 121°C for 30 min. Thereafter, 70 ml of sterilized water is added.* Monascus purpureus *(KCCM: Korean Culture Center for Microorganisms, Seoul, Korea) is inoculated into the seed culture medium, and it incubated for 5 days with shaking (150 rpm) at 30°C. Finally, after sterilizing the mixture of* Artemisia argyi* H. extract (400 mL) and rice (28 g), 2% of seeded strains are inoculated in sterilized mixture, and then it incubated for 5 days with shaking (150 rpm) at 30°C. And then, this extraction was lyophilized using a vacuum-tray freeze dryer (Operon, Gimpo, Korea). The lyophilized sample (extraction yield: 4.2%) was ground to powder form and stored at −20°C.

### 2.3. Neuronal Cell Culture and Measurement of Neuronal Cell Protect Effect

PC 12 cells (KCLB 21721; Korea Cell Line Bank, Seoul, Korea) were incubated in RPMI-1640 (Gibco BRL, Grand Island, NY, USA) medium at 37°C under 5% CO_2_.

DCF-DA forms formazan (fluorescence DCF) by intracellular reactive oxygen species (ROS) such as H_2_O_2_. Cells (1 × 10^6^/well) were cultured in 96-well plates for 24 h, and then sample was treated into the well. After 24 h, cells were manipulated with or without 200 *μ*M H_2_O_2_. And then the 50 *μ*M DCF-DA dissolved in phosphate buffered saline (PBS) was treated into the well. To measure fluorescence, fluorescence microplate reader (Infinite 200, Tecan Co., San Jose, CA, USA) with 485 nm excitation and 530 nm emission filters was used [11].

The MTT reduction assay was used to measure neuroprotection of AAFM against oxidative stress induced by hydrogen peroxide. After AAFM or vitamin C (positive control) treatment on PC12 cells (10^4^ cells/well on 96-well), the cells passed through the preculturing process for 48 h. 200 *μ*M hydrogen peroxide was treated into the cell for 3 h without control group. And then, MTT formazan produced by the mitochondria in living cells was measured using a microplate reader (Bio-Rad, CA, USA) with the test wave length of 570 nm and the reference wavelength of 690 nm

The LDH assay was used to verify nerve cell membrane protection of AAFM. The measurement of endoenzyme such as LDH amount released into the medium can be used to evaluate the degree of nerve cell membrane damage. The LDH content was measured with the LDH kit of Sigma-Aldrich Chemical Co.

### 2.4. Animals Design

All experiments were conducted strictly pursuant to the guidelines which were set by the Animal Care and Use Committee at Gyeongsang National University under the (certificate: GNU-131105-M0067) and performed in accordance with the Policy of the Ethical Committee of Ministry of Health and Welfare, Republic of Korea. The Institute of Cancer Research (ICR) mice (4 weeks old, male) were purchased from the Samtako (Osan, Korea), and mice were housed in cages under maintained conditions (12 h light/dark cycle, 55% humidity, and 23–25°C) and allowed free access to food and water. Mice were randomly assigned to five groups: control (water-oral administration + saline-injection) group, TMT (water-oral administration + TMT-injection) group, and AAFM (AAFM-oral administration + TMT-injection) groups (5, 10, and 20 mg/kg of body weight, resp.). The AAFM was dissolved in drinking water, and it was fed once a day for 3 weeks. After a period of 3 weeks, the TMT (7.1 *μ*g/kg of body weight) was dissolved in 0.85% sodium chloride solution (w/v), and TMT (100 *μ*L) was intraperitoneally given to each mouse except for the control group. Control group was intraperitoneally injected sodium chloride solution (100 *μ*L) without TMT.

### 2.5. Behavioral Tests of Mice

Three days after the TMT-injection, Y-maze test was conducted. The maze was made of plastic painted black. Each arm was 15 cm high, 10 cm wide, and 33 cm long, and they were set with a certain angle from each other. The test started by placing a mouse at the end of an arm and allowing it to freely move during 8 min. Each entry was measured when a mouse completely placed its feet, and the movement of the mouse at the arm entries was recorded using a Smart 3.0 Video tracking system (Panlab, Barcelona, Spain). Alternation is defined as entries into the three arms in an overlapping triplet set. In order to calculate the alternation behavior, the ratio of actual alternation to possible alternation was multiplied by actual alternation/(total number of arm entries − 2) × 100 (%).

The passive avoidance test box consists of two zones, as a light zone and a dark zone. In the learning period, mice were initially placed in the light zone and after mice were familiarized. After 1 min, the guillotine door between the two chambers was opened. And then when the mice entered the dark zone, mice were provided with an electric shock (0.5 mA, 3 s). After 24 h, the latency time in light zone was measured [12].

The Morris water maze test according to Morris [13] was slightly modified before its practice. A stainless steel circular pool (90 cm in diameter) was divided into four sections (S, W, E, and N zones) with random visible cue on the walls. To make it opaque, squid ink (Cebesa, Valencia, Spain) was added to the water. And the temperature was kept at 22 ± 2°C. In the middle of the W zone, a platform (6 cm in diameter) was placed without any change in its position during the whole process of training. The latency time of each mouse's escape from the water onto the platform was recorded up to 60 seconds as the maximum. The training sessions (days 1–4) were carried out with four trials on a daily basis for consecutive days. The platform was eliminated in a probe test (day 5), and then the mice had to swim for 60 s and the time spent in the W zone was measured (using Smart 3.0 video tracking system).

### 2.6. Measurement of Acetylcholinesterase (AChE) Activity and Acetylcholine (Ach) Content from the Mice Brain

The mixed whole brains homogenate (5 *μ*L) with 65 *μ*L of sodium phosphate buffer (50 mM, pH 8.0) was incubated at a fixed temperature, 37°C, for 15 min. As soon as an Ellmans's reaction mixture [70 *μ*L; 0.5 mM acetylthiocholine and 1 mM 5, 50-dithio-bis (2-nitrobenzoic acid)] in a 50 mM sodium phosphate buffer (pH 8.0) was added to the above reaction mixture and absorbance was read as 405 nm.

The level of ACh was determined in accordance with the method described by Vincent et al. [14]. The brain homogenate and alkaline hydroxylamine reagent [2 M hydroxylamine in HCl and 3.5 N sodium hydroxide] react at room temperature for 1 min. 0.5 N HCl and 0.37 M FeCl_3_ (in 0.1 N HCl) were added and absorbance was measured at 540 nm.

### 2.7. Measurement of Oxidative Stress and Antioxidant System in Mice Brain

Homogenates (brain with PBS) were centrifuged at 400*g* (for 10 min at 4°C). 1x Cell Extraction Buffer [10% SOD buffer, 0.4% (v/v) Triton X-100, and 200 *μ*M Phenylmethanesulfonyl fluoride in ethanol] is added in pellets and then incubated on ice for 30 min. The mixtures were centrifuged at 10,000 ×g for 10 min at 4°C and the supernatant is used to measure SOD levels using SOD assay kit.

Brain with 5% metaphosphoric acid homogenates centrifuged at 14,000 ×g for 15 min at 4°C. The supernatant was treated to 2 M 4-vinylpyridine and incubated for 1 h at room temperature. And oxidized GSH and GSH were measured using commercial kits.

Brain homogenates with PBS were centrifuged (6000 ×g for 10 min at 4°C), and the supernatant was mixed with 0.67% TBA solution with 1% phosphoric acid and then incubated in a water bath (95°C) for 1 h. After cooling, absorbance was measured at 532 nm.

### 2.8. Measurement of Mitochondrial Activity from the Mice Brain

According to the procedure of Dragicevic et al., whole brains were homogenized with 5 volumes of isolation buffer containing 215 mM mannitol, 75 mM sucrose, 0.1% bovine serum albumin (BSA, Bioworld Dublin OH, USA), 1 mM EGTA, and 20 mM HEPES (pH 7.2) to separate the mitochondria and centrifuged at 1,300 ×g for 5 min [15]. The supernatant was centrifuged once more at 13,000 ×g for 10 min. After the supernatant was eliminated, the pellet was added with isolation buffer containing 0.1% digitonin in DMSO. After 5 min, isolation buffer was added and the pellet was centrifuged at 13,000 ×g for 15 min. Afterwards, the pellets were resuspended using the isolation buffer without ethylene glycol tetraacetic acid (EGTA) and then centrifuged at 10,000 ×g for 10 min. Finally, isolation buffer without EGTA was added to the pellet and adopted to the experiment.

Measurement of mitochondrial ROS production was conducted with DCF-DA assay [11]. 25 *μ*M DCF-DA was added to the isolated mitochondria for 20 min, and then fluorescent production was quantified with a fluorescent (excitation filter 485/20 nm, emission filter 528/20 nm) fluorescence reader.

In order to measure the membrane potential of isolated mitochondria, after mixing 20 *μ*L of the mitochondria with assay buffer [isolation buffer without 5 Mm EGTA with pyruvate and 5 mM malate], a solution of 1 *μ*M 5,5′,6,6′-tetrachloro-1,1′3,3′,-tetraehylbenzimi-dazolycarbocyanine iodide (JC-1) in DMSO was reacted, and, then, the mixture was stirred gently at room temperature for 20 min in the dark. It was measured with a fluorescent (excitation 530/25 nm, emission 590/35 nm) microplate reader.

### 2.9. Western Blot Analysis

Brain tissues were added with ProtinEx™ Animal cell/tissue (GeneAll Biotechnology, Seoul, Korea) and 1% protease inhibitor cocktails (Thermo Fisher Scientific, Rockford, IL, USA) and centrifuged at 13,000*g* for 10 min at 4°C. After segregating the protein using sodium dodecyl sulfate polyacrylamide gel electrophoresis (SDS-PAGE), it was transferred to a polyvinylidene difluoride (PVDF) membrane (Millipore, Billerica, MA, USA). The membranes were blocked with 5% nonfat dry milk in Tris-Buffered Saline (TBS) with 0.1% of Tween 20 (TBST) buffer. After 1 h, primary antibodies were diluted (1 : 1000) in a dilute solution (0.1% sodium azide and 0.5% BSA in TBST). After incubating diluted primary antibodies with the membrane under gentle agitation overnight, the membrane was washed 3 times (10 min each time) in TBST. And then, after allowing the secondary antibody solution to react with the membrane for 1 h, it was washed again. Finally, prior to detecting the luminescence with Chemi-doc (Korea Biomics, Seoul, Korea), the membrane was exposed to an enhanced chemiluminescence reagent. The density of the band was analyzed with Image J Software (National Institutes of Health, Bethesda, MD, USA). The protein was calculated using density of target protein/density of *β*-actin as a loading control.

### 2.10. Ultra-Performance Liquid Chromatography Quadrupole-Time-of-Flight (UPLC-Q-TOF) MS Analysis

UPLC Q-TOF/MS was used to analyze the quality of main compounds in the AAFM. An electrospray source in negative ion mode was used to obtain MS and MS/MS data. Phenolic compounds were separated on an ACQUITY UPLC BEH C18 with oven temperature at 40°C and a flowing rate of 0.3 mL/min. The following shows how a linear solvent gradient of binary mobile phase during analysis was applied: 99% A/1% B at 0–10 min. The conditions for MS analyses included the drying gas (N_2_) temperature at 35°C, fragmentor voltage at 175 V, nebulizer pressure at 45 psi, drying gas flow at 10 L/min, capillary voltage at 4000 V, and mass range from* m*/*z* 100 to 1000.

### 2.11. Statistical Analysis

All data were expressed as mean ± SD. The different capital letters from “A” represent statistical difference (*p* < 0.05) of each group in a high order using Duncan's new multiple-range test of SAS ver. 9.1 (SAS Institute Inc., Cary, NC, USA).

## 3. Results and Discussion

### 3.1. Effect of AAFM on Neuronal Cell Protective Effect

Neuronal cells have weak structural characteristics against oxidative stress because they have relatively high amounts of unsaturated fatty acids. Therefore, ROS can result in changes in the integrity and fluidity of the cell membrane, and increased oxidative stress is related to neurodegenerative diseases such as AD [10, 16].

**Figure 1:**
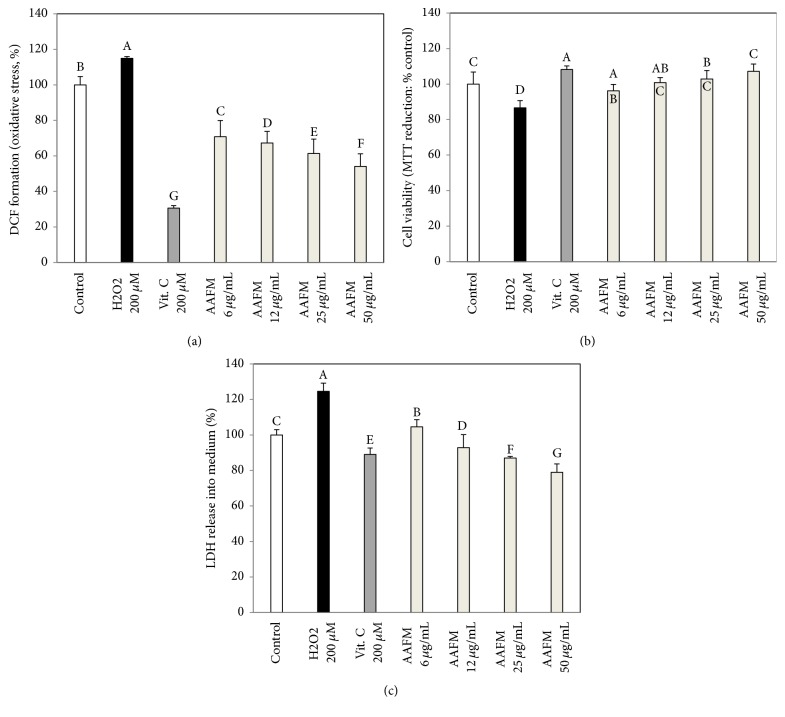
Neuronal cell protective effects of AAFM on H_2_O_2_ induced oxidative stress in PC12 cells. DCF-DA assay (a), MTT assay (b), and LDH assay (c) were examined. Data shown represent means ± SD (*n* = 8). The different capital letters from “A” represent statistical difference (*p* < 0.05) of each group in a high order.

To measure the protective effect of AAFM against H_2_O_2_-induced oxidative stress, DCF-DA assays were carried out to confirm ROS production. The intracellular ROS level of the H_2_O_2_ group was increased to 115.0 ± 4.7% (*p* < 0.05) compared with that of the control group (100.0 ± 4.7%) (Figure 1(a)). However, the intracellular ROS level of the AAFM group significantly decreased compared with that of the H_2_O_2_ group. In addition, according to the results of the MTT assay, the H_2_O_2_ group (86.6 ± 4.4%) (*p* < 0.05) had lower cell viability than the control (100.0 ± 6.7%), vitamin C (108.2 ± 2.0%) (*p* < 0.05), and AAFM (6 *μ*g/mL; 96.2 ± 3.6%, 12 *μ*g/mL; 100.8 ± 2.8%, 25 *μ*g/mL; 102.9 ± 4.7%, 50 *μ*g/mL; 107.1 ± 4.2%) groups (Figure 1(b)). Moreover, in the LDH assay, the LDH release of the H_2_O_2_ group (124.6 ± 4.6%) (*p* < 0.05) was increased compared with that of the control group (100 ± 3.0%) (Figure 1(c)). In addition, compared with the H_2_O_2_ group, the vitamin C group (89.0 ± 3.6%) (*p* < 0.05) showed decreased LDH release. AAFM also had an excellent inhibitory effect on LDH release into the medium by protecting the neuronal cell membranes. In other words, AAFM protected neuronal cells from H_2_O_2_-induced oxidative stress.

These results showed that the antioxidant activity of the phenolic compounds from AAFM protected the cells against H_2_O_2_-induced oxidative stress. In addition, according to a previous study, when* Monascus purpureus* is fermented with rice, secondary metabolites, such as dimerumic acid and deferricoprogen, are generated, and these secondary metabolites can protect the neuron against oxidative stress through their antioxidant activity [17]. Therefore, the neuronal protective effect of AAFM was produced through the interaction of secondary metabolites by the fermentation and phenolic compounds of AAFM.

### 3.2. Improving Effect of AAFM on TMT-Induced Cognitive Dysfunction

Y-maze and passive avoidance tests were carried out to assess spatial cognition and short-term memory abilities, respectively. The Morris water maze test is a means of evaluating experimental animals' spatial memory learning ability and long-term memory ability [12, 13].

**Figure 2:**
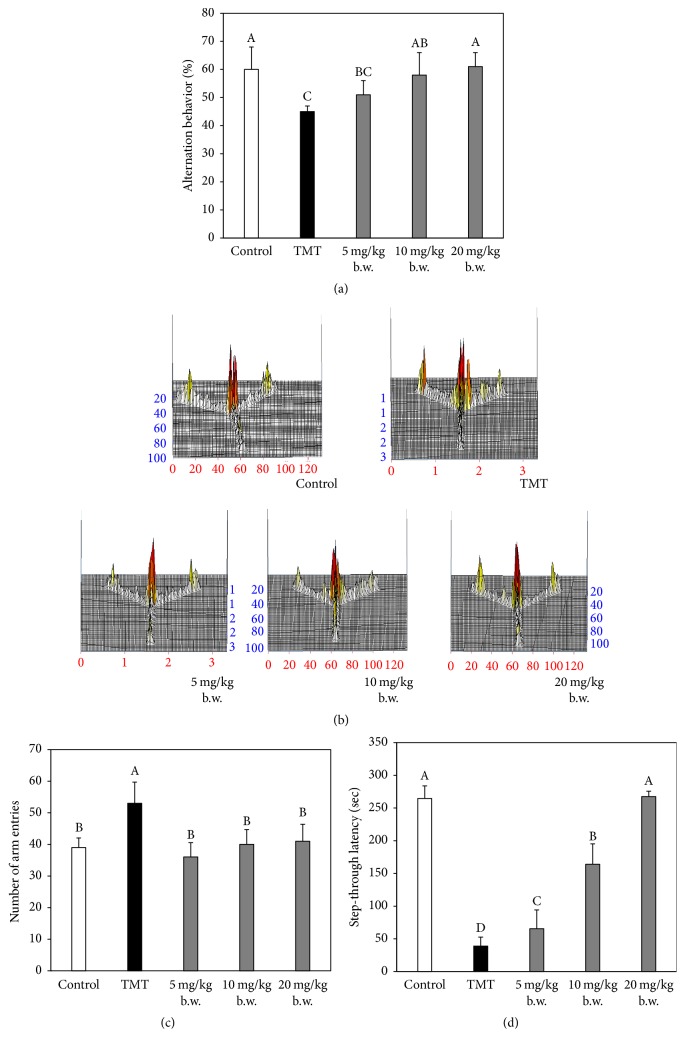
Effect of AAFM on Y-maze and passive avoidance tests. Spontaneous alteration behavior (a), path motion (b), number of arm entries (c) in Y-maze test, and step-through latency (d) in passive avoidance test. Data shown represent means ± SD (*n* = 8). The different capital letters from “A” represent statistical difference (*p* < 0.05) of each group in a high order.

**Figure 3:**
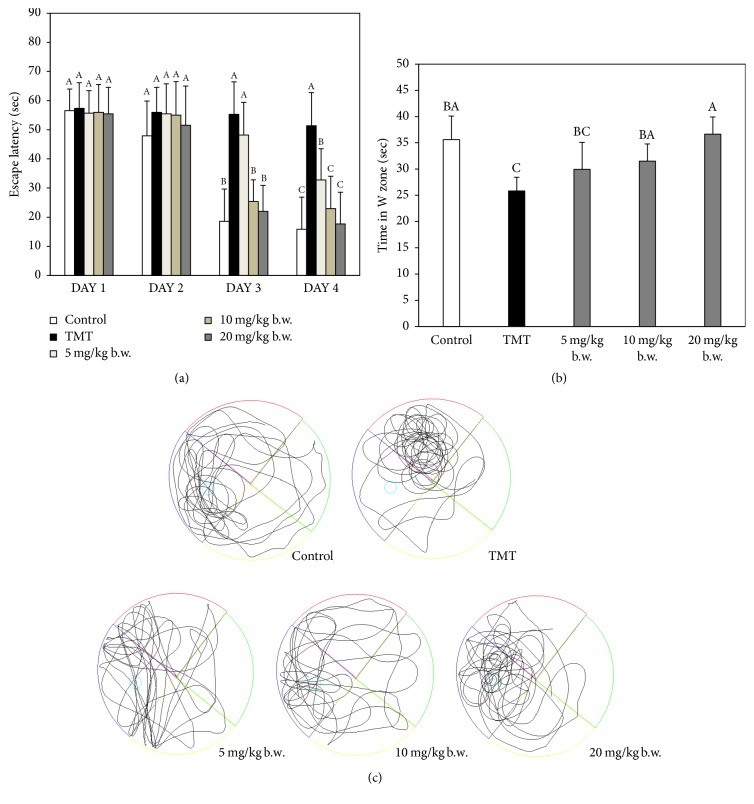
Effect of AAFM on escape latency in the training trial (a) and probe test (b) and path of motion in probe test (c). Data shown represent means ± SD (*n* = 8). The different capital letters from “A” represent statistical difference (*p* < 0.05) of each group in a high order.

The results of the behavioral tests are shown in Figures 2 and 3. In the Y-maze test, the TMT group (45 ± 2%) (*p* < 0.05) showed reduced spatial cognition abilities in comparison with the control group (60 ± 8%). On the other hand, the AAFM 5, 10, and 20 mg/kg of body weight groups showed results of 51 ± 5%, 58 ± 8%, and 61 ± 5%, respectively (Figure 2(a)). According to previous research, animals exposed to TMT exhibit cognitive impairment as well as hyperactivity disorder due to severe TMT-induced hippocampal damage [18]. In our results, the TMT group also showed hyperactivity compared with the control group. However, the AAFM group was confirmed to have effectively improved TMT-induced hyperactivity disorder (Figures 2(b) and 2(c)).

The passive avoidance test was conducted to assess short-term memory of stress, as shown in Figure 2(d). The latency time of the TMT group (38.8 ± 13.7 s) (*p* < 0.05) was reduced compared to that of the control group (264.5 ± 19.3 s). The AAFM groups showed improved memory dysfunction compared to that of the TMT group.

Finally, the result of the Morris water maze test is shown in Figure 3. During the four days of repeating the experiment (hidden trial), the TMT group did not reduce escape latency significantly, while the control and AAFM groups did (Figure 3(a)). In the probe test (fifth day), the platform was removed, and, then, the latency time in the W zone was measured to examine spatial and long-term memory ability. The latency time in the W zone of the TMT group (25.8 ± 2.6%) (*p* < 0.05) was decreased in comparison with that of the control group (35.6 ± 4.5%), and those of the AAFM 5, 10, and 20 mg/kg of body weight groups were 30.0 ± 5.1%, 31.5 ± 3.3%, and 36.7 ± 3.3%, respectively (Figure 3(b)). Movement in the probe test is shown in Figure 3(c).

In conclusion, TMT-induced cognitive impairment improved as the concentration of AAFM increased. In particular, the AAFM 20 mg/kg of body weight group showed an outstanding improvement effect compared to that of the control group, and these results showed that AAFM had a protective effect against TMT-induced cognitive dysfunction.

### 3.3. Effect of AAFM on ACh Level and AChE Activity

ACh, a neurotransmitter found in the brain, tends to be reduced in the hippocampus by various causes, including oxidative stress in the cerebral cortex. ACh is decomposed by AChE in the cholinergic system at the end of the neuron, and it is reported that an excessive increase of AChE activation can trigger the decomposition of ACh related to a decrease of cognitive function [19].

**Figure 4:**
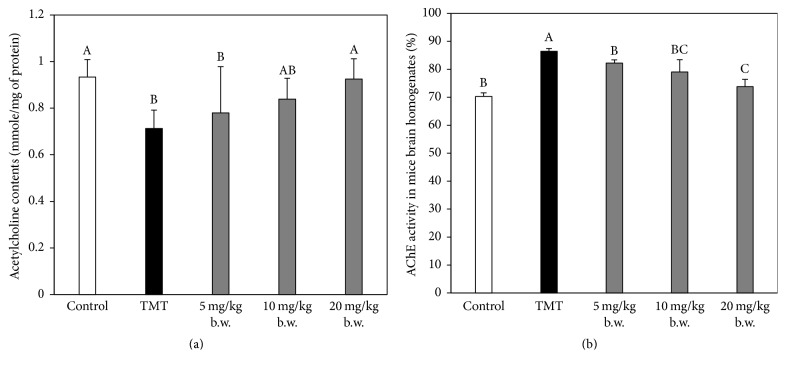
Effect of AAFM on ACh contents (a), and AChE activity (b) in mice brain homogenates. Data shown represent means ± SD (*n* = 8). The different capital letters from “A” represent statistical difference (*p* < 0.05) of each group in a high order.

After* in vivo* tests, brain tissues were extracted to measure the ameliorating effect of the cholinergic system. In this study, the ACh content of the TMT group (0.71 ± 0.08 mmol/mg of protein) (*p* < 0.05) was decreased compared with that of the control group (0.93 ± 0.08 mmol/mg of protein). The ACh contents of the AAFM 5, 10, and 20 mg/kg of body weight groups were 0.77 ± 0.2 mmol/mg of protein (*p* < 0.05), 0.83 ± 0.09 mmol/mg of protein, and 0.93 ± 0.09 mmol/mg of protein (Figure 4(a)), respectively. An increase of AChE activity was found in the TMT group (115.8 ± 5.6%) (*p* < 0.05) compared with the control group (100 ± 4.9%), and more effective suppression was found in the AAFM groups (5 mg/kg of body weight: 103.2 ± 7.3%, 20 mg/kg of body weight: 95.2 ± 6.6%, 20 mg/kg of body weight: 90.9 ± 6.1% (*p* < 0.05)) compared with the TMT group (Figure 4(b)). As a result of this study, we confirmed the reduction of ACh content and the increase of AChE activity in TMT injected mice. However, in the AAFM groups, the ACh content increased and the AChE activity decreased. According to the report of Nag and De, methanolic extracts of fruits (*Terminalia chebula, Terminalia bellirica, *and* Emblica officinalis*) have AChE inhibitory activity because of phenolic compounds like gallic acid, ellagic acid, and so on [20]. In addition, in recent research, it is confirmed that several natural polyphenols have antiacetylcholinesterase activity [21]. Therefore, these results suggest that AAFM also has polyphenols having antiacetylcholinesterase activity, and thus we think that the anticholinergic activity of AAFM's polyphenols was accompanied by improvement of cognitive functions, like learning and memory in TMT-induced cognitive dysfunction mice. Consequently, AAFM could become a candidate substance as an AChE inhibitor for treating dementia.

### 3.4. Effect of AAFM on SOD Content, Level of Oxidized GSH/Total GSH Ratio, and MDA Level

Antioxidant enzymes, such as SOD and GSH, neutralize oxidation phenomena. However, when the amount of free radicals generated exceeds such enzymes' neutralization capacity, neuronal ageing and degenerative deformation occur rapidly [22].

**Figure 5:**
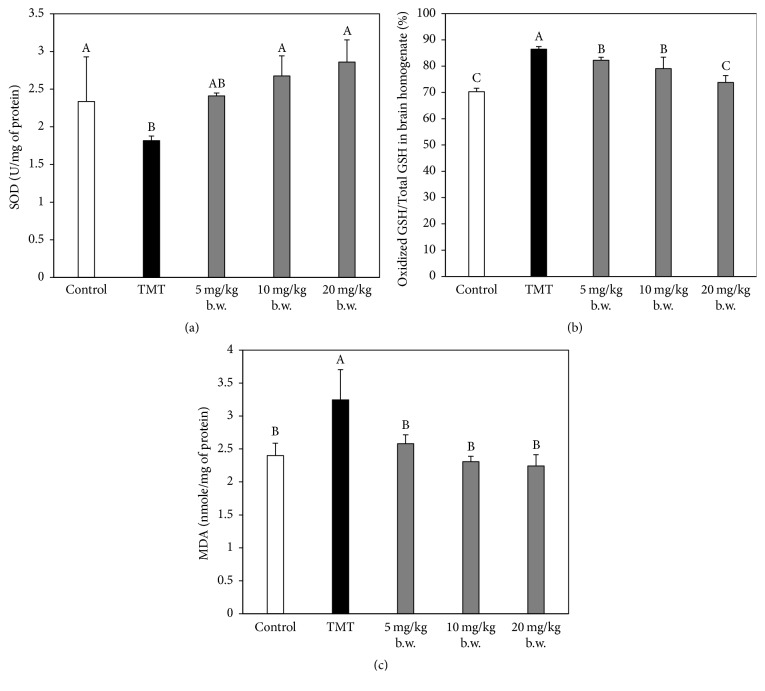
Effect of AAFM on SOD contents (a), MDA contents (b), and oxidized GSH/total GSH ratio (c) from TMT-induced defective mice brain homogenates. Data shown represent means ± SD (*n* = 5). The different capital letters from “A” represent statistical difference (*p* < 0.05) of each group in a high order.

TMT leads to neuronal oxidative damage and the extended elevation of ROS levels [8]. Therefore, in our study, the TMT group (1.82 ± 0.06 U/mg of protein) showed decreased SOD levels compared to that of the control group (2.34 ± 0.59 U/mg of protein). Conversely, the AAFM 5, 10, and 20 mg/kg of body weight groups showed increased SOD levels (2.41 ± 0.04 U/mg of protein, 2.67 ± 0.27 U/mg of protein, and 2.86 ± 0.30 U/mg of protein, resp.) (Figure 5(a)).

GSH is an important antioxidant enzyme, and the oxidized GSH/total GSH ratio is usually used as an indicator of oxidative stress [23]. Accordingly, as expected, the oxidized GSH/total GSH ratio in the TMT group (86.5 ± 1.0%) (*p* < 0.05) was higher than that of the control group (70.3 ± 1.3%). Moreover, in the AAFM groups (5 mg/kg of body weight: 82.3 ± 1.1% (*p* < 0.05), 10 mg/kg of body weight: 79.1 ± 4.4% (*p* < 0.05), and 20 mg/kg of body weight: 73.8 ± 2.6%), the oxidized GSH/total GSH ratios were lower than that of the TMT group (Figure 5(b)). In addition, MDA contents resulted in a similar pattern to that found in the analysis result of the oxidized GSH/total GSH ratio, because MDA is an indicator of lipid peroxidation caused by oxidative stress (Figure 5(c)). Since brain tissue is composed of numerous polyunsaturated fatty acids carrying out various signaling functions, it becomes vulnerable to lipid peroxidation, and lipid peroxidation in the brain is an indicator of mild cognitive impairment [24]. As a result, the TMT-induced damage of the antioxidant system increased MDA levels and caused cognitive dysfunction. According to report of Ebrahimi and Schluesener, the most prominently discussed effect of polyphenols is their antioxidant activity [21]. In other words, antioxidant effects of AAFM's polyphenols could reduce the TMT-induced oxidative stress. In conclusion, AAFM could protect the collapse of the antioxidant system by reducing oxidative stress. Accordingly, AAFM was able to mitigate learning difficulties and memory impairments caused by TMT-induced oxidative stress.

### 3.5. Effect of AAFM on Mitochondrial Activity

Mitochondria are the fundamental organelles that act to control ageing and age-related neurodegeneration by adjusting cellular energy status and ROS and ATP production [25]. Mitochondrial abnormalities can cause improper energy production as well as abnormalities in calcium homeostasis and the abnormal excretion of calcium ions in cells. In addition, they can activate the apoptosis pathway in neuronal cells by releasing cytochrome C (Cyto. C). Deterioration of the mitochondria membrane potential (MMP) resulting from ROS caused by abnormal mitochondrial activity can impair energy efficiency and cause cellular apoptosis, which can ultimately lead to cell death [25, 26].

**Figure 6:**
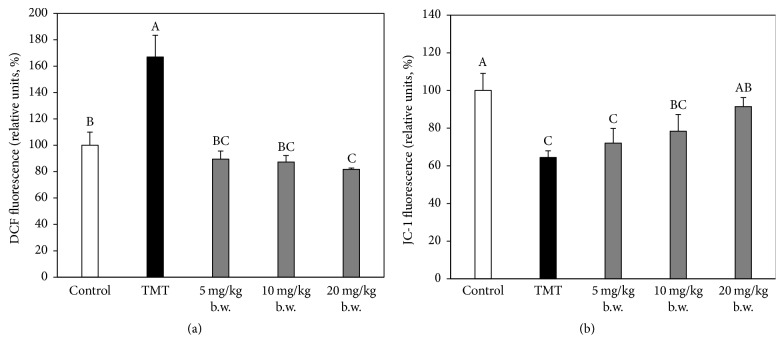
Effect of AAFM on mitochondrial function of brain tissue in TMT injected mice. ROS production (a) and mitochondria membrane potential (MMP) (b). Data shown represent means ± SD (*n* = 5). The different capital letters from “A” represent statistical difference (*p* < 0.05) of each group in a high order.

In comparison to the control group (100 ± 10.1%), the TMT group (167.0 ± 16.4%) (*p* < 0.05) showed an increase in mitochondrial ROS production in our experimental results. On the other hand, mitochondrial ROS production of the AAFM groups (5 mg/kg of body weight: 89.5 ± 6.1%, 10 mg/kg of body weight: 87.4 ± 4.9%, and 20 mg/kg of body weight: 81.7 ± 1.1% (*p* < 0.05)) was improved from TMT toxicity (Figure 6(a)). Accordingly, the MMP level of the TMT group (64.5 ± 3.5%) (*p* < 0.05) was decreased compared with that of the control group (100.0 ± 9.1%) because of the increased mitochondrial ROS production. Moreover, as predicted, the MMP levels of the AAFM groups (5 mg/kg of body weight: 72.0 ± 7.8% (*p* < 0.05), 10 mg/kg of body weight: 78.4 ± 8.8% (*p* < 0.05), and 20 mg/kg of body weight: 91.4 ± 4.7%) were higher than that of the TMT group.

According to Wiegman et al., mice exposed to ozone, a source of oxidative stress, showed decreased mitochondrial membrane potential, increased mitochondrial oxidative stress, and reduced mitochondrial complex I, III, and V expression [27]. In other words, oxidative stress and mitochondrial dysfunction are closely related. Therefore, we conclude that the effect of AAFM on the reduction of oxidative stress by TMT led to the protection of mitochondrial function, and ROS production was reduced in mitochondria. For this reason, TMT-induced learning and memory deficits could be mitigated through AAFM treatment.

### 3.6. Effect of AAFM on Apoptotic Proteins

Changes in apoptotic signaling molecules were analyzed by western blot assay to assess the effect of AAFM, and an experiment was performed using the brain tissue of the control, TMT, and AAFM 20 mg/kg of body weight groups, because the AAFM 20 mg/kg of body weight group showed the best effect of the three AAFM groups.

In terms of the improvement of neurodegenerative disorders, Akt plays an important role as a prosurvival kinase. The phosphorylation of Akt as the activated form inactivates the preapoptotic proteins, such as glycogen synthase kinase-3*β* (GSK-3*β*), Bcl-2-associated death promoter (BAD), and caspase 9, by phosphorylating these proteins, and promotes cell longevity pathways [28].

**Figure 7:**
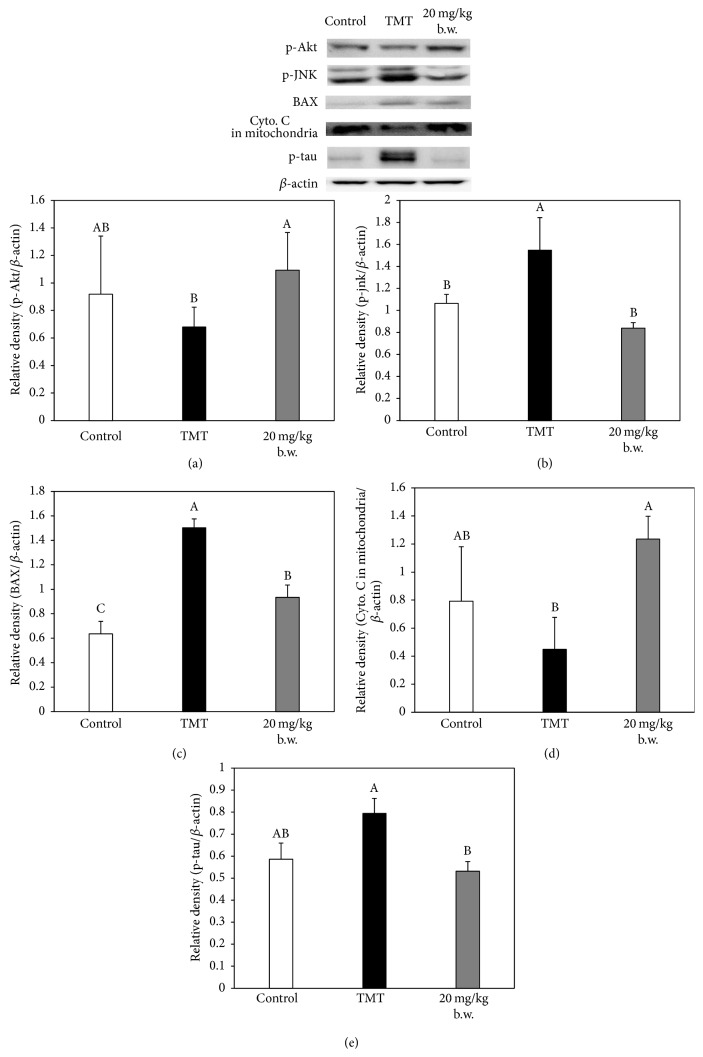
Effect of AAFM on the expression of apoptotic signaling molecules in TMT injected mice brain. p-Akt/*β*-actin (a), p-JNK/*β*-actin (b), BAX/*β*-actin (c), cytochrome C in mitochondria/*β*-actin (d), and p-tau (e). Data shown represent means ± SD (*n* = 6). The different capital letters from “A” represent statistical difference (*p* < 0.05) of each group in a high order.

In contrast, c-Jun N-terminal kinase (JNK) stimulates the activation of proapoptotic proteins, and the activation of JNK occurs through the dual phosphorylation of tyrosine (Tyr) and threonine (Thr) residues [29]. Therefore, the activation of Akt and inactivation of JNK are important factors for cell survival. In the results, the p-Akt expression of the TMT group (relative density: 0.68 ± 0.14) was dramatically decreased compared with that of the control group (relative density: 0.92 ± 0.42). On the other hand, the p-Akt expression of the AAFM 20 mg/kg of body weight group (relative density: 1.09 ± 0.27) was higher than that of the TMT group as expected. In addition, the expression of p-JNK was shown to be the opposite of the expression of p-Akt in each group (relative density of control, TMT, and AAFM 20 mg/kg of body weight groups: 1.06 ± 0.08, 1.55 ± 0.30 (*p* < 0.05), and 0.84 ± 0.05, resp.) (Figures 7(a) and 7(b)).

In addition, as mentioned above, when BAD is phosphorylated by p-Akt, it forms the BAD-(14-3-3) protein heterodimer. Bcl-2 and Bcl-xL, which are antiapoptotic proteins, are activated, while BAX/Bak-triggered apoptosis is inactivated. BAX/Bak also leads to a loss in membrane potential by interacting with the mitochondrial voltage-dependent anion channel. Accordingly, Cyto. C is released from the mitochondria to the cytosol [30]. For these reasons, the TMT group (relative density of BAX and Cyto. C in mitochondria: 1.50 ± 0.07 (*p* < 0.05) and 0.45 ± 0.23, resp.) showed increased expression of BAX and release of Cyto. C in the mitochondria compared with the control group (relative density of BAX and Cyto. C in mitochondria: 0.64 ± 0.1 and 0.79 ± 0.39, resp.). On the other hand, the AAFM 20 mg/kg of body weight group (relative density of BAX and Cyto. C in mitochondria: 0.93 ± 0.10 (*p* < 0.05) and 1.24 ± 0.16, resp.) showed better results than the TMT group (Figures 7(c) and 7(d)).

In addition, p-Akt also inactivates GSK-3*β*, a type of tau protein kinase I (TPKI). Tau protein kinases I and II (TPKI and TPKII) are candidate enzymes responsible for the hyperphosphorylation of tau, and TPKII strongly enhances the action of TPKI. The hyperphosphorylation of tau induced by GSK-3*β* activity leads to neuronal cell death; accordingly, it is closely related to AD [28, 31]. As a result of measuring the amount of p-tau, we confirmed that the p-tau level of the TMT group (relative density: 0.79 ± 0.07) was increased compared with that of the control group (relative density: 0.59 ± 0.07), while the p-tau level was decreased in the AAFM 20 mg/kg of body weight group (relative density: 0.53 ± 0.04) (Figure 7(e)).

To sum up, AAFM effectively inhibited apoptosis through Akt activation and JNK inactivation, and it is thought that the repression of apoptosis in neuronal cells by AAFM could lead to the improvement of cognitive function. In addition, this result may be the result of mitochondrial dysfunction; therefore we confirmed again that AAFA is effective in ameliorating mitochondrial dysfunction.

### 3.7. UPLC Q-TOF/MS Analysis of AAFM

**Figure 8:**
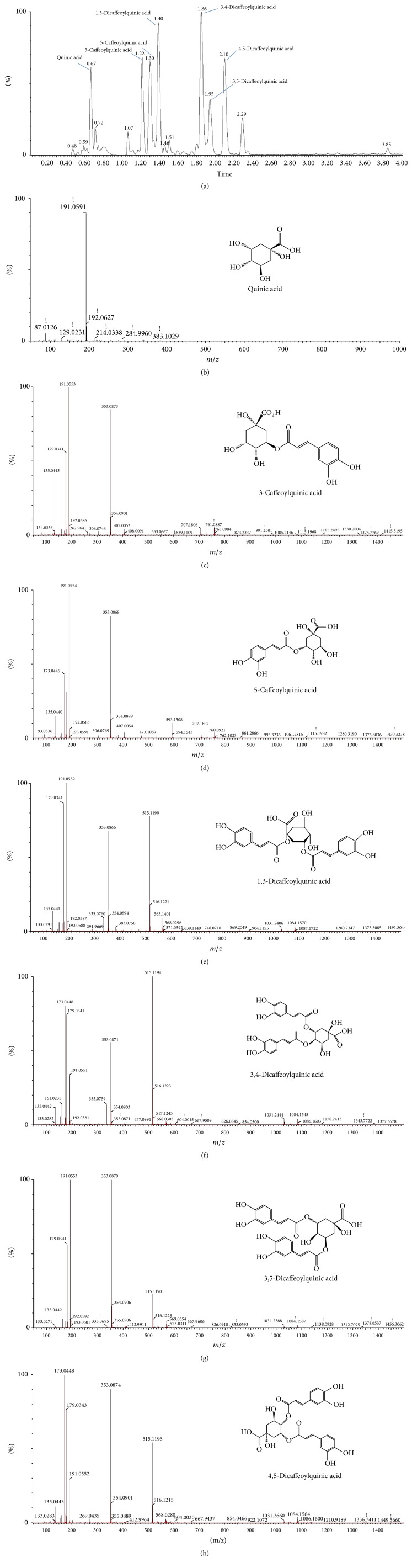
UPLC Q-TOF/MS chromatography in negative ion mode (a) and MS^2^ spectra of quinic acid (b), 3-Caffeoylquinic acid (c), 5-Caffeoylquinic acid (d), 1,3-Dicaffeoylquinic acid (e), 3.4-Dicaffeoylquinic acid (f), 3.5-Dicaffeoylquinic acid (g), and 3.5-Dicaffeoylquinic acid (h).

Phytochemicals of AAFM were identified by UPLC-Q-TOF MS analysis using an MS^2^ scan with mass fragmentation. These phytochemicals in AAFM were identified as seven major peaks (retention time at 0.67, 1.22, 1.30, 1.40, 1.86, 1.95, and 2.10 min). When the results of each MS^2^ scan were compared with references, the main phytochemicals of AAFM were confirmed to be derivatives of quinic acid (compound 1: Quinic acid, compound 2: 3-Caffeoylquinic acid, compound 3: 5-Caffeoylquinic acid, compound 4: 1.3-Dicaffeoylquinic acid, compound 5: 3,4-Dicaffeoylquinic acid, compound 6: 3.5-Dicaffeoylquinic acid, and compound 7: 4,5- Dicaffeoylquinic acid) (Figure 8), and 3.4-Dicaffeoylquinic acid and 1.3-Dicaffeoylquinic acid were identified as major phenolic compounds [32, 33].

Previous research confirmed that* Artemisia annua* L., which also belongs to the genus* Artemisia*, also has quinic acid derivatives as the major constituents. Moreover, 3.5-Dicaffeoylquinic acid was identified as a major component of* Artemisia annua* L. in an LC–negative ion ESI-MS total ion current (TIC) profile [33]. In our previous research, 3.5-Dicaffeoylquinic acid was analyzed by a Q-TOF MS system in ESI-negative mode and found to be a main phenolic compound of* Artemisia argyi *H. ethyl acetate fractions [34]. In other words, these results suggest that the physiological activity could be affected by components changed by* Monascus purpureus* fermentation. However, the summative or synergistic effect of secondary metabolites produced during microbial fermentation on the antiamnesic effect could not be confirmed, and this will be investigated in the near future.

In investigations of the analyzed substances, it has been reported that quinic acid derivatives have neuroprotective effects against oxidative stress through their antioxidant activity [35]. In particular, it has been reported that chlorogenic acid has an ameliorating effect on scopolamine-induced amnesia via the inhibition of AChE and antioxidant activity in mice [36]. Consequently, the effect of AAFM on TMT-induced cognitive dysfunction has been considered to be improved by the quinic acid derivatives produced by microbial fermentation.

## 4. Conclusion

The improvement effect of AAFM on TMT-induced cognitive dysfunction in mice was confirmed. AAFM showed a superior protective effect against H_2_O_2_-induced oxidative stress in neuronal cells. In an animal behavior test, AAFM intake improved learning and memory abilities in mice with TMT-induced cognitive impairment. According to the results of the brain tissue analysis in the AAFM groups, oxidative stress and the reduction of ACh were ameliorated. AAFM prevented the occurrence of mitochondrial dysfunction caused by TMT-induced oxidative stress, and, thus, apoptosis was inhibited. These physiological activities were assumed based on the quinic acid derivatives in* Artemisia argyi* H. under liquid-state fermentation by* Monascus purpureus. *Consequently, the administration of AAFM could lead to the improvement of cognitive function. In addition, we confirmed that phenolic compounds produced by microbial fermentation could be changed by various strains used for fermentation; accordingly, the physiological activity of the fermented product could be affected.

## Supplementary Material

Supplementary Material 1 shows polyphenol contents of extracts fermented by various strains. In this result, the extract fermented by *Monascus purpureus* had the highest polyphenol contents compared to other strains.
Supplementary Material 2 shows antioxidant activity(ABTS radical scavenging activity) of extracts fermented by various strains. In this result, the extract fermented by *Monascus purpureus* had the best antioxidant activity compared to other strains.
Supplementary Material 3 shows acetylcholinesterase inhibitory activity of extracts fermented by various strains. The AChE inhibition activity of extract fermented by *Lactobacillus brevis* was the highest compared to other strains, but there was no significant differences from the extract fermented by *Monascus purpureus*. Therefore, in our experiment, the extract fermented by *Monascus purpureus* was used to investigate.
