# Supplementary material for: Cognitive Function of Artemisia argyi H. Fermented by Monascus purpureus under TMT-Induced Learning and Memory Deficits in ICR Mice
Source: Evid Based Complement Alternat Med. 2017 Sep 10;2017:5809370. doi: 10.1155/2017/5809370 (PMC5610803; doi:10.1155/2017/5809370)
Supplement: Supplementary file 1 — Supplementary Material 1 shows polyphenol contents of extracts fermented by various strains. In this result, the extract fermented by Monascus purpureus had the highest polyphenol contents compared to other strains. Supplementary Material 2 shows antioxidant activity(ABTS radical scavenging activity) of extracts fermented by various strains. In this result, the extract fermented by Monascus purpureus had the best antioxidant activity compared to other strains. Supplementary Material 3 shows acetylcholinesterase inhibitory activity of extracts fermented by various strains. The AChE inhibition activity of extract fermented by Lactobacillus brevis was the highest compared to other strains, but there was no significant differences from the extract fermented by Monascus purpureus. Therefore, in our experiment, the extract fermented by Monascus purpureus was used to investigate. [file 5809370.f1.docx]

- Supplementary Material 1 shows polyphenol contents of extracts fermented by various strains. In this result, the extract fermented by *Monascus purpureus* had the highest polyphenol contents compared to other strains.


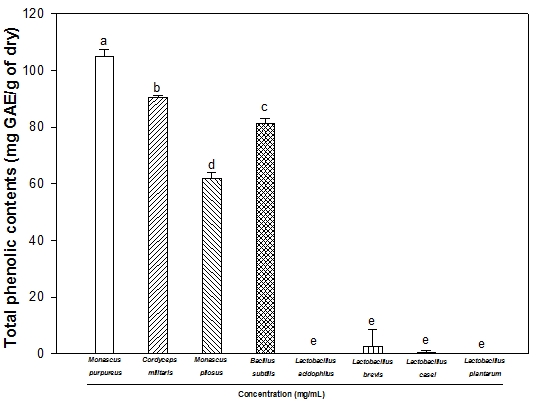


**Figure 1. Total phenolic contents of extract from *Artemisia argyi* H. under liquid-state fermention by various strains**. Data shown represent means ± SD (n=3). Statistical differences were represented using different small letters (𝑝< 0.05).

- Supplementary Material 2 shows antioxidant activity(ABTS radical scavenging activity) of extracts fermented by various strains. In this result, the extract fermented by *Monascus purpureus* had the best antioxidant activity compared to other strains.


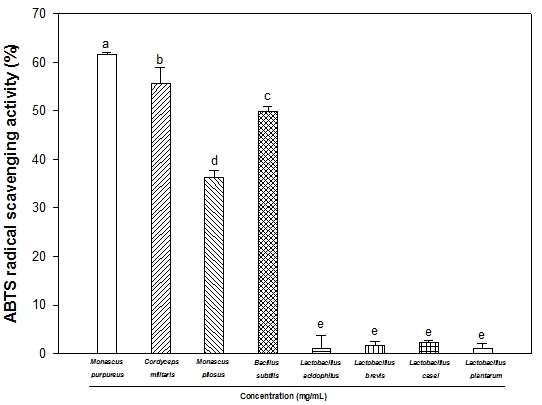


**Figure 2. ABTS radical scavenging activity of extract from *Artemisia argyi* H. under liquid-state fermention by various strains**. Data shown represent means ± SD (n=3). Statistical differences were represented using different small letters (𝑝< 0.05).

- Supplementary Material 3 shows acetylcholinesterase inhibitory activity of extracts fermented by various strains. The AChE inhibition activity of extract fermented by *Lactobacillus brevis* was the highest compared to other strains, but there was no significant differences from the extract fermented by *Monascus purpureus*. Therefore, in our experiment, the extract fermented by *Monascus purpureus* was used to investigate.


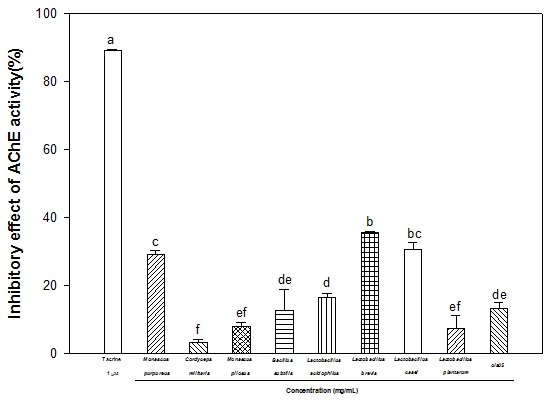


**Figure 3. Effect of *Artemisia argyi* H. fermented by various strains on AChE activity compared with tacrine as positive control.** Data shown represent means ± SD (n=3). Statistical differences were represented using different small letters (𝑝< 0.05).
